# Concussion and Sport: Progress is Evident

**DOI:** 10.1007/s40279-022-01713-z

**Published:** 2022-06-20

**Authors:** Philip J. O’Halloran, Anthony P. Kontos, Michael W. Collins

**Affiliations:** 1 Department of Neurosurgery, Queen Elizabeth Hospital, Birmingham, UK; 2 School of Physiotherapy, The Royal College of Surgeons in Ireland, Dublin, Ireland; 3 UPMC Concussion Network, Waterford, Ireland; 4 UPMC Sports Medicine Concussion Program, Department of Orthopaedic Surgery, University of Pittsburgh, Pittsburgh, PA USA

Dear Editor,

Mild traumatic brain injuries or concussions are a substantial health concern, particularly in collision and contact sports such as rugby and football. Consequently, there is growing concern regarding the acute and chronic effects of repeated brain trauma, with player welfare protocols central to these discussions. Athletes, parents and patient advocacy groups have voiced concerns over the management and safeguarding of athletes during their respective playing careers from youth sport to professional levels. However, the science related to the purported long-term effects of repeated brain trauma in athletes is still in its infancy, and has struggled to keep pace with the growing concern and calls to action.

Recently, a spate of rule changes and protocols were introduced across many sporting codes including rugby, soccer, Gaelic Football and Australian Football, in an attempt to improve player safety. Although these changes are well intentioned and supported by biomechanical studies, only time will tell if they will be effective on the field. In the meantime, fear and concern among athletes and parents may result in decreased sport participation to avoid potential long-term effects. Researchers have reported a mismatch between the restoration of physical symptoms and physiological homeostasis in acute and chronic concussion [1], as well as injury to white matter tract fibres [2] which represents a period of vulnerability to athletes. The work of the current authors has highlighted the adverse effects, including a doubling of recovery time and evidence for a dose response associated with continued sport participation immediately following a concussion [3, 4]. Specifically, athletes who played for just 15 min beyond the point of having symptoms took on average 25–28 days longer to recover than players who were removed from play immediately [4]. Together, these findings highlight the risks of returning to play prematurely as well as the importance of early recognition of concussion and immediate removal from play. Specifically, players with a suspected concussion should not continue to play, even for a few minutes, as they risk more serious injury and prolonged recovery. Recently, the Rugby Football League and the Australian Football League introduced a revised 11-day and 12-day return-to-play (RTP) protocol, respectively, in response to concerns about returning to play too soon. Despite these efforts, players continue to place themselves at risk by under-reporting, concealing or ignoring symptoms. However, the news on concussion is not all bad …

Recent advancements in multi-domain assessments including neurocognitive, oculomotor and vestibular, as well as advances in the application of bio-engineering, neuroimaging, serum/saliva biomarkers and translational research to concussion offer hope for a future of evidence-based clinical care. For example, computerised neurocognitive testing, which measures memory, reaction time and processing speed, is a valid and reliable method for identifying cognitive deficits following a concussion [5, 6]. In fact, athletes were 17.2% less likely to RTP following a concussion when neurocognitive testing was performed compared to when it was not performed [7]. These findings highlight the importance of a multi-domain assessment that includes symptom, cognitive, ocular, vestibular and post-exertion evaluations, rather than relying on only athlete-reported symptoms to inform RTP decision making. Similarly, the Vestibular/Ocular Motor Screening (VOMS) tool, which evaluates vestibular and oculomotor symptoms and impairment, has demonstrated the ability to identify concussion [8] and predict prolonged recovery [9]. The results from these and other assessments help clinicians to make more informed and evidence-based decisions regarding treatments and subsequent RTP. However, outdated “one size fits all” clinical approaches to treatment involving predominantly rest continue to persist and do not address the heterogeneous nature of this injury.

Fortunately, active rehabilitation approaches that target specific symptoms and impairment are now recognised as a key component of clinical care following concussion. Time following injury may play a role in the nature and structure of these symptoms [10]. Additionally, recent evidence suggests that the timing of specialty care for concussion is a strong predictor of recovery time [11, 12]. Strong evidence exists demonstrating the benefits of active rehabilitation in this cohort of patients [13], with a recent randomised controlled trial illustrating a 48% reduction in post-concussive symptoms in an aerobic exercise group versus stretching exercises [14]. This injury can generate a variety of symptoms that necessitate different treatment strategies. Identifying the involved symptoms and impairment using a multi-domain assessment allows clinicians to target treatments to individual athletes with more successful outcomes [15]. Modifiable domains such as vestibular, ocular, cognitive, headache/migraine and anxiety/mood represent validated predictors of a sports-related concussion [5], thereby allowing individualised therapeutic approaches and subsequent RTP in a controlled/safe manner. The authors recently reported evidence from a randomised trial supporting the effectiveness of precision vestibular rehabilitation for adolescents following concussion [15].

In this era of precision medicine, the role of the multi-disciplinary team is vital to conducting a multi-domain assessment, and providing the optimum personalised treatment plan in collaboration with the athlete. The combined expertise of disciplines including neurosurgery, neuropsychology, neurology, primary care, physiotherapy and other allied health subspecialties represents the ideal model of care to effectively evaluate and treat athletes with concussion today and into the future.
